# There Is No Such Thing as a Psychiatric Disorder/Disease/Chemical Imbalance

**DOI:** 10.1371/journal.pmed.0030318

**Published:** 2006-07-25

**Authors:** Fred Baughman

**Affiliations:** **1**El Cajon, CaliforniaUnited States of America

In her recent *PLoS Medicine* article, Christine Phillips writes: “ADHD [attention deficit hyperactivity disorder] joins dyslexia and glue ear as disorders that are considered significant primarily because of their effects on educational performance” [ 1]. A “disorder” is “a disturbance of function, structure, or both,” and thus, the equivalent of an objective abnormality/disease [ 2]. In neurologically normal children, dyslexia cannot be proved to be a disorder/disease. “Glue ear,” however, is otitis media, an objective abnormality/ disease. Phillips continues: “In the case of ADHD, there has been a complex, often heated debate in the public domain about the verity of the illness,” but proceeds, without an answer, to consider “the roles of teachers as brokers for ADHD and its treatment.”

In 1948, “neuropsychiatry” was divided into “neurology,” dealing with diseases, and “psychiatry,” dealing with emotions and behaviors [ 3]. If there is a macroscopic, microscopic, or chemical abnormality, a disease is present. Nowhere in the brains or bodies of children said to have ADHD or any other psychiatric diagnosis has a disorder/disease been confirmed. Psychiatric drugs appeared in the fifties. Psychiatry and the pharmaceutical industry authored the “chemical imbalance” market strategy: they would call all things psychological “chemical imbalances” needing “chemical balancers”—pills.

At the September 29, 1970, hearing on Federal Involvement in the Use of Behavior Modification Drugs on Grammar School Children, Ronald Lipman of the United States Food and Drug Administration (FDA), argued: “hyperkinesis is a medical syndrome. It should be properly diagnosed by a medical doctor” [ 4].

In 1986, Nasrallah et al. [ 5] reported brain atrophy in adult males treated with amphetamines as children, concluding: “since all of the HK/MBD [hyperkinetic/minimal brain dysfunction] patients had been treated with psychostimulants, cortical atrophy may be a long-term adverse effect of this treatment.”

At the 1998 National Institutes of Health (NIH) Consensus Development Conference on ADHD, Carey [ 6] stated: “The ADHD behaviors are assumed to be largely or entirely due to abnormal brain function. The DSM-IV does not say so but textbooks and journals do.... What is now most often described as ADHD...appears to be a set of normal behavioral variations.”

However Swanson and Castellanos [ 7], having reviewed the structural magnetic resonance imaging (MRI) research, testified: “Recent investigations provide converging evidence that a refined phenotype of ADHD/HKD (hyperkinetic disorder) is characterized by reduced size in specific neuroanatomical regions of the frontal lobes and basal ganglia.” I challenged Swanson, asking: “Why didn't you mention that virtually all of the ADHD subjects were on stimulant therapy—the likely cause of their brain atrophy?” [ 8] Swanson confessed this was so—that there had been no such studies of ADHD-untreated cohorts.

The Consensus Conference Panel concluded: “We do not have a valid test for ADHD... there are no data to indicate that ADHD is a brain malfunction” [ 9]. (This wording appeared in the version of the final statement of the Consensus Conference Panel distributed at the press conference in the final part of the Consensus Conference, November 18, 1998. This wording, which appeared for an indeterminate time on the NIH Web site, was subsequently removed and replaced with wording claiming “validity” for ADHD.)

In 2002, Castellanos et al. [ 10] published the one and only MRI study of an ADHD-untreated group. However, because the ADHD-untreated patients were two years younger than the controls, the study was invalid, leaving stimulant treatment, not the never-validated disorder, ADHD, the likely cause of the brain atrophy.

In 2002, Daniel Weinberger, of the National Institute of Mental Health, claimed “major psychiatric diseases” are associated with “subtle but objectively characterizable changes” but could reference not a single proof (quoted in [ 11]).

In 2002, the Advertisement Commission of Holland [ 12] determined that the claim that ADHD is an inborn brain dysfunction was misleading and enjoined the Brain Foundation of the Netherlands to cease such representations.

In 2003, Ireland prohibited GlaxoSmithKline from claiming that the antidepressant Paxil “works by bringing serotonin levels back to normal.” Wayne Goodman of the FDA acknowledged that claims that selective serotonin reuptake inhibitors correct a serotonin imbalance go “too far,” but had the temerity to suggest that “this is reasonable shorthand for expressing a chemically or brain-based problem” (quoted in [ 13]).

At an FDA hearing on March 23, 2006, I testified: “Saying any psychiatric diagnosis ‘is a brain-based problem and that the medications are normalizing function’ is an anti-scientific, pro-drug lie” [ 14]. Yet this has become standard practice throughout medicine, for example, at the American Psychiatric Association [ 15], American Medical Association [ 16], American Academy of Child and Adolescent Psychiatry, American Academy of Pediatrics, Child Neurology Society, American Academy of Family Physicians [ 17], FDA [ 13], and virtually all US government health-care agencies.

Journal articles [ 6], press releases, ads [ 18], drug inserts, and research informed consent documents say, or infer, that psychological diagnoses are abnormalities/diseases. All patients and research participants with psychological problems are led to believe they have an abnormality/disease, biasing them in favor of medical interventions, and against nonmedical interventions (e.g., love, will power, or talk therapy), which presume, as is the case, that the individual is physically and medically normal and without need of a medical/pharmaceutical intervention.

The FDA is the agency most responsible for conveying the facts needed by the public to make risk versus benefit and informed consent decisions. Instead—by protecting industry, not the public—the FDA is a purveyor of the psychiatric “disease” and “chemical imbalance” lie. This must change.
